# PAH Contamination, Sources and Health Risks in Black Soil Region of Jilin Province, China

**DOI:** 10.3390/toxics12120937

**Published:** 2024-12-23

**Authors:** Guzailinuer Aihemaitijiang, Lujuan Zhang, Mingtang Li, Yanan Chen, Jiquan Zhang, Feng Zhang, Chunli Zhao

**Affiliations:** 1 College of Resources and Environment, Jilin Agricultural University, Changchun 130118, China; 19842831983@163.com (G.A.); zlj20012559@163.com (L.Z.); limtdoc2008@163.com (M.L.); chenyn061@nenu.edu.cn (Y.C.); 2 College of Jilin Management, Changchun Institute of Technology, Changchun 130012, China; 3 Institute of Natural Disaster Research, School of Environment, Northeast Normal University, Changchun 130024, China; zhangjq022@nenu.edu.cn; 4 College of Forestry and Grassland Science, Jilin Agricultural University, Changchun 130118, China

**Keywords:** black soil, agriculture soil, polycyclic aromatic hydrocarbons, human health risk assessment, source identification

## Abstract

Soils in the Black Soil Zone of northeast China are experiencing pollution from polycyclic aromatic hydrocarbons (PAHs) as the region undergoes urbanization. In this study, 119 topsoil samples were collected from the black soil agricultural area in Jilin Province, China to investigate the characteristics and spatial distribution of 16 PAHs. The total concentration of ∑16 PAHs in the agricultural soils ranged from 2.546 to 33.993 mg/kg, with a mean value of 9.99 mg/kg. Positive matrix factorization (PMF) analysis indicated that vehicle exhaust and oil combustion were identified as the main contributors to traffic- and energy-related pollution. The inherited lifetime carcinogenicity risk (ILCR) was found to be relatively low, indicating a low potential risk in this region, with adults (1.34 × 10^−5^) exhibiting a higher risk than adolescents (8.62 × 10^−6^) and children (7.49 × 10^−6^). The highest values for intake, skin contact, and inhalation routes were observed in the adult group, suggesting that adult residents in certain areas may be at increased health risk. This study enhances our understanding of the pathways through which PAHs enter agricultural soils in Jilin Province and provides insights that could aid in addressing PAH pollution in black soil, ultimately contributing to more sustainable agricultural practices in the region.

## 1. Introduction

Globally, there has been a significant increase in the severity of soil contamination due to accelerated urbanization, industrial changes, unsustainable resource utilization, wastewater irrigation, air pollution, and excessive use of fertilizers and pesticides [1,2]. Among the numerous pollutants, the contamination of polycyclic aromatic hydrocarbons (PAHs) in agricultural soils is becoming an increasingly serious issue and represents a significant global environmental concern [3]. PAHs form a class of aromatic compounds composed of at least two fused benzene rings, and they tend to accumulate in soil [4]. These compounds have been identified as carcinogenic, teratogenic, and mutagenic [5]. PAHs can enter the human body through the food chain [6,7]. The 16 representative PAHs (∑16PAHs) are classified as priority pollutants in both China and the United States, and there is a documented link between PAHs and various diseases, including heart disease, inflammation, gastrointestinal issues, and lung cancer [8]. Furthermore, evidence suggests that the long-term consumption of foods containing PAHs may significantly increase the risk of cancer [9,10]. PAHs can enter the soil through various pathways, including atmospheric deposition. Consequently, soil has emerged as a significant source of PAHs [11,12]. PAHs are highly toxic and tend to bioconcentrate, leading to alterations in soil particle size, porosity, and water-holding capacity, which can jeopardize the diversity of microbial populations [13]. Furthermore, exposure to PAHs on crop surfaces has the potential to affect the human immune system, ultimately resulting in adverse health effects [14]. Given these considerations, it is evident that the contamination of agricultural soils by PAHs has garnered considerable public attention. Therefore, it is crucial to assess the extent of PAH contamination in agricultural soils and the associated ecological and health risks.

Soil plays a pivotal role in advancing agriculture, significantly influencing the quality and ecological functionality of the surrounding environment. The contamination of agricultural soils by PAHs is on the rise [15,16]. Industrial pollution and the increased use of agrochemicals have introduced numerous hazardous substances, particularly organic pollutants, into agricultural soils [17]. PAHs, resulting from pyrolysis or incomplete combustion, represent a major environmental pollutant associated with agricultural soil contamination [18]. Understanding the sources of PAHs is essential for the effective management of these pollutants. The impact of agricultural soils on PAH contamination depends on both the soil characteristics and the potential sources of contamination [19]. Prior research has categorized the origins of PAHs in soil into two distinct groups: natural and anthropogenic [20]. Natural sources encompass plants, microorganisms, natural fires, and the release of PAHs from fossil fuels [21]. In contrast, anthropogenic sources primarily arise from the incomplete combustion or pyrolysis of various fossil fuels, wood, paper, and other hydrocarbon-containing materials [22]. Previous research has shown that the combustion of coal and biomass can lead to soil contamination with PAHs [23]. The volume of PAHs originating from human activities has increased in recent years, paralleling the shift towards more industrialized and urbanized societies.

China is the world’s most significant source of PAHs, which are widely dispersed across the atmosphere, surface water, soil, and sediments [24]. From 2000 to 2018, total PAH concentrations in urban soils in China ranged from 65.01 to 23,603.05 μg/kg, with a mean concentration of 801.98 μg/kg; notably, 47% of the country is severely contaminated, while only 13% remains uncontaminated [16,25]. China’s ’dual-carbon strategy’ is anticipated to influence the distribution of PAH emissions in agricultural soils. Researchers have discovered that this strategy could potentially reduce PAH pollution in China’s topsoil [26]. Researchers reviewed PAH concentrations in Chinese soils, reporting that the 16 PAHs exhibited the following concentrations across different regions: northeastern China (1467 μg/kg) > northern China (911 μg/kg) > eastern China (737 μg/kg) > southern China (349 μg/kg) > northwestern China (209 μg/kg) [27]. Soil contamination in the northeast is particularly severe, and PAH contamination represents a significant challenge. The black soil region in northeastern China is a crucial source of food and oil, playing a vital role in ensuring national food security [28]. Black soil is a valuable natural resource, characterized by a high organic matter content and a dark coloration. Its soil matrix is primarily loess-like clay, flood deposits, alluvial deposits, moraines and wind deposits, and other loose sediments. It has a favourable character, high fertility, and is suitable for farming. It is therefore imperative that further study into black soil be conducted.

Jilin Province is located in the central region of northeast China. The area cultivated with black soil spans 4.6 × 10^7^ hm^2^, accounting for 65.5% of the province’s total cultivated area and 24.82% of the cultivated area within the Northeast Black Soil Zone. This latter statistic indicates a significant proportion of black soil in the northeast region. Jilin Province, recognized as one of China’s key old industrial bases, has a substantial demand for energy, with coal consumption reaching approximately 110 million tons annually [29]. The combustion of large quantities of coal directly contributes to the emission of solid particles, which significantly affects the surrounding soil environment. Black soils are crucial for food security due to their fertility and capacity for high food production. However, the condition of black soils is deteriorating as agricultural practices intensify and climate conditions worsen [30,31]. In recent years, these soils have faced the dual challenges of declining fertility and agrochemical pollution. Consequently, it is essential to ensure the ecological security of black soil regions, which can be achieved through research into the contamination of PAHs in these soils.

Currently, soil pollution by PAHs is a significant issue both domestically and internationally. Historically, research on black soils has predominantly concentrated on enhancing fertility, with less emphasis on PAH contamination. Jilin Province, a key region of black soil in northeastern China, has a lengthy history of heavy industry and rapid urbanization. However, investigations into PAHs in the black soil of this region remain scarce. This study focused on black soil in Jilin Province with the following primary research objectives: (1) to quantify the concentrations of 16 distinct PAHs in the soil; (2) to examine the relationship between PAH levels and soil properties; (3) to evaluate the potential risks associated with PAHs in Jilin’s black soil; and (4) to identify the sources of PAHs.

## 2. Materials and Methods

### 2.1. Study Area Description

Jilin Province is located in the central region of northeast China and features a temperate continental monsoon climate, characterized by four distinct seasons [32]. The climate in Jilin Province is notably complex, exhibiting a transition from humid to semi-humid and then to semi-arid conditions as one moves from the southeast to the northwest. The annual temperature range is between 35 °C and 42 °C, with a daily variation of 10 °C to 14 °C. The region enjoys an average of 2259 to 3016 h of sunshine annually and receives average annual precipitation ranging from 400 to 600 mm [33]. The topography of Jilin Province features a slope from the southeast to the northwest, where the eastern region is characterized by mountainous and hilly terrain, while the central area consists predominantly of plains.

### 2.2. Sample Acquisition

**Figure 1:**
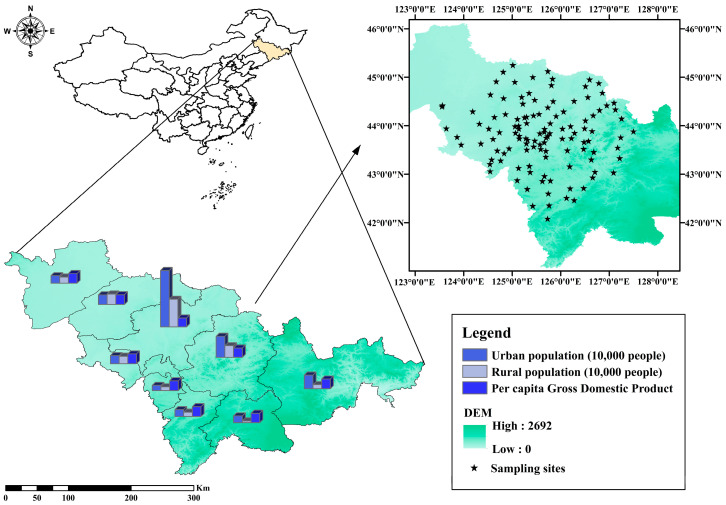
Sampling locations of 119 arable soils in the study area.

A total of 119 samples were collected using the plum sampling method in the typical black soil area of Jilin Province (Figure 1). The samples were collected at a depth of 0–20 cm into the topsoil. The samples were combined into separate portions and placed into clean, self-sealing polyethylene bags within the laboratory of the Chinese Games. Following the removal of debris, the soil was subjected to a 2 mm sieving process and subsequently frozen in advance of the analytical phase. The initial weight of the samples was recorded as 1–2 kg. The locations of the sampling points were determined using the global positioning system (GPS) throughout the course of the sampling process.

### 2.3. Main Reagents and Materials

Determination was undertaken of the following 16 polycyclic aromatic hydrocarbons: naphthalene (Nap), acenaphthylene (Acy), acenaphthene (Ace), fluorene (Flu), phenanthrene (Phe), anthracene (Ant), fluoranthene (Flt), pyrene (Pyr), benz[a] anthracene (BaA), chrysene (Chr), benzo[b]fluoranthene (BbF), benzo [k]fluoranthene (BkF), benzo[a]pyrene (BaP), indeno[1,2,3-cd]pyrene (IND), dibenz[a,h]anthracene (DahA) and benzo[g,h,i]perylene (BghiP). Organic solvents, such as acetone, n-hexane and dichloromethane, are chromatographically pure grade. The main instruments were as follows: florisil extraction column, 1 g/6 mL; GC-MS; ultrasonic extractant; rotary evaporator; and nitrogen blower (Organomation Inc., Berlin, MA, USA). The PAH mixture contained 16 congeners (200 μg/mL), with acenaphthene deuterium (ANA-d10) and phenanthrene deuterium (PHE-d10) as internal standards (SUPELCO, Bellefonte, PA, USA).

### 2.4. Soil Tests and Extracting the PAHs

Determination of soil organic matter (SOM), pH and soil moisture content (SMC), was undertaken as follows. We weighed 10.0 g of dried soil samples through a 2 mm sieve in a 50 mL beaker and added 25 mL water. We sealed the bottle with plastic wrap and stirred for 2 min, it was then left for 30 min to determine the pH. A 0.2 g air-dried soil sample passing through a 0.149 mm sieve was weighed and the organic matter content was determined using the potassium dichromate method.

PAHs in Jilin black soil were measured using an ultrasonic washer and gas chromatography mass spectrometry (GC-MS, Clarus 680/600 T, PerkinElmer Inc., Waltham, MA, USA). We weighed 10 g of soil and placed it in a Pyrex tube with a 60-mesh sieve. An amount of 40 μg of the quantitative substitute standard was added and we subsequently performed ultrasonic extraction using 60 mL of an acetone: hexane mixed solution (1:1, *v/v*). We set the ultrasonic instrument to 20 °C for 30 min, then centrifuged the sample three times. We transferred the extract to a round-bottomed flask. The extract was concentrated to 2mL using a rotary evaporator at 45 °C. The concentrated solution was purified via passage through a magnesium silicate column, with 2 g of copper powder added to the top layer of the column to effectively eliminate sulfur compounds. Then, the internal standard was added and the eluate was concentrated to 1 mL with nitrogen. The PAH concentration was then measured using GC-MS. The gas inlet was set to 270 °C, the no-shunt mode was set to on, and the injection volume was 1.0 μL. The column flow was 1.0 mL/min. The temperature was increased from 20 °C/min to 80 °C for 2 min, then from 10 °C/min to 290 °C for 10 min. Before determining the concentration of PAHs, it is necessary to generate calibration curves. The measured concentrations were processed and analyzed using SPSS and origin software.

### 2.5. Quality Control

The implementation of laboratory blanks, blank spiking, matrix spiking, and other quality control measures within the laboratory setting ensures the reliability and accuracy of analytical results. It is crucial that the quality control requirements for the entire procedure ensure that blank samples do not exceed the method detection limit of the target analytes. A standard solution of 16 PAHs was utilized at a concentration of 200 μg/mL, and clean glassware, analytical-grade chemicals, and high-purity deionized water were employed to prevent sample contamination and confirm the absence of interferences prior to use. The target compounds were not detected in the laboratory blank. The internal standard technique was applied to quantify and identify compounds based on the retention times of the standards. The levels of PAHs recovered from the soil samples ranged from 81% to 118%.

### 2.6. Risk Assessment of PAHs

Of the 16 priority control PAHs identified by the Environmental Protection Agency (EPA), 7 chemicals have been classified as carcinogenic. Among these, benzo[a]pyrene (BaP) is recognized as one of the most toxic PAHs [34]. The toxicity of individual PAH compounds varies considerably, making it inappropriate to simply sum the total amounts of each monomer in the evaluation. BaP is predominantly produced during the incomplete combustion of organic materials and is widely distributed in the environment [35]. In risk assessments associated with PAHs, BaP is typically used as the standard reference point, with toxicity equivalence factors (TEFs) calculated according to the methodology outlined in the following equation:

(1) $${TEQ}_{BaP}=\sum (C_{i}\times {TEF}_{i}),$$

where C_i_ represents the concentration of compound i, while TEF_i_ denotes the toxicity equivalence factor of compound i relative to BaP. The toxicity equivalence factors for the 16 PAH monomers in relation to BaP are as follows: 0.001, 0.001, 0.001, 0.001, 0.001, 0.010, 0.001, 0.001, 0.100, 0.010, 0.100, 0.100, 1.000, 0.100, 1.000, and 0.010 [36].

The Incremental Lifetime Carcinogenic Risk (ILCR) model is a health risk assessment framework proposed by the U.S. Environmental Protection Agency (EPA) for evaluating the health risks associated with polycyclic aromatic hydrocarbons (PAHs) in soils [37,38]. Generally, the ILCR model is utilized to assess the cancer risk of PAHs through three primary exposure routes: oral ingestion, respiratory inhalation, and dermal contact [39]. The general population of Jilin Province can be categorized into distinct age groups: children (0–10 years), adolescents (11–18 years), and adults (19–70 years). The carcinogenic risk of PAHs via various exposure routes is calculated using Equations (2)–(5), as follows:

(2) $${ILCR}_{ing}=\frac{CS\times \left({CSF}_{ing}\times \sqrt[{3}]{\left(BW/70\right)}\right)\times {IR}_{ing}\times EF\times ED}{BW\times AT\times 10^{6}},$$

(3) $${ILCR}_{der}=\frac{CS\times \left({CSF}_{der}\times \sqrt[{3}]{\left(BW/70\right)}\right)\times SA\times AF\times ABS\times EF\times ED}{BW\times AT\times 10^{6}},$$

(4) $${ILCR}_{inh}=\frac{CS\times \left({CSF}_{inh}\times \sqrt[{3}]{\left(BW/70\right)}\right)\times {IR}_{inh}\times EF\times ED}{BW\times AT\times PEF},$$

(5) $${ILCR}_{s}={ILCR}_{ing}+{ILCR}_{der}+{ILCR}_{inh},$$

where ILCR_ing_, ILCR_der_ and ILCR_inh_ are health risk values due to oral ingestion, dermal contact and inhalation, respectively; CS is the total toxic equivalence of the 16 PAHs relative to BaP (Equation (1)), (μg/kg); CSF is a carcinogenic slope factor for inhalation exposure to BaP, CSF_ing_, CSF_der_, CSF_inh_ is 7.3, 25, 3.85 (mg/kg/d), respectively [40,41]; BW is body weight (kg), values are 16, 45 and 70 for children, adolescents and adults, respectively [12]; AT represents the average life time (d) of the carcinogen and takes the value 25,550 [42]; EF is exposure frequency (d/year) and takes the value of 350 [43]; ED stands for exposure duration (a), which takes values of 6, 14 and 30 for children, adolescents and adults, respectively [44]; SA is exposed skin surface area (cm^2^/d), which takes values of 2800, 2800 and 5700 for children, adolescents and adults, respectively [45]; AF is the dermal adherence factor (mg/cm^2^) [40] and takes values of 0.2, 0.2 and 0.07 for children, adolescents and adults, respectively [40]; IR_ing_ is soil ingestion rate (mg/d), with values of 100, 100 and 200 for children, adolescents and adults, respectively [41]. IR_inh_ stands for air inhalation rate (m^3^/d) and takes values of 10.9, 17.7 and 17.5 for children, adolescents and adults, respectively [40]. ABS stands for the dermal absorption factor and has a value of 0.13. PEF is the particle emission factor (m^3^/kg) and has a value of 1.36 × 10^6^ [40]. ILCRs represent the total risk for each of the three exposure routes; IRing stands for soil ingestion rate (mg/d), with values of 100, 100 and 200 for children, adolescents and adults, respectively.

### 2.7. Source Apportionment

Identifying the sources of polycyclic aromatic hydrocarbons (PAHs) in the black soil region of Jilin Province is crucial for assessing risks and preventing pollution. This study employed the characteristic ratio method and the positive matrix factorization (PMF) model for source identification.

#### 2.7.1. Feature Ratio Method

The characteristic ratio method leverages the differences in thermal stability among isomers to identify the sources of polycyclic aromatic hydrocarbon (PAH) emissions. Ratios between various components can be utilized to ascertain the potential emission sources of PAHs in soils within Jilin Province. The following ratios were selected for diagnostic analyses in this study: Flt/(Flt + Pyr), Ant/(Ant + Phe), IND/(IND + BghiP), BaA/(BaA + Chr), and Pyr/BaA. Results indicating low molecular weight (LMW) PAHs to high molecular weight (HMW) PAH ratios of less than 1 suggest emissions from burning, while ratios greater than 1 indicate oil sources. Specifically, Ant/(Ant + Phe) ratios of less than 0.1 are indicative of oil sources, whereas values exceeding 0.1 suggest burning sources. Similarly, Flt/(Flt + Pyr) ratios of less than 0.4 point to oil sources, ratios between 0.4 and 0.5 correspond with liquid fossil fuel combustion sources, and ratios greater than 0.5 are associated with coal or biomass combustion sources [46]. For BaA/(BaA + Chr), ratios less than 0.2 indicate oil sources, ratios between 0.2 and 0.35 suggest mixed sources, and ratios exceeding 0.35 are indicative of burning sources [47]. Lastly, IND/(IND + BghiP) ratios of less than 0.2 are associated with oil sources, ratios between 0.2 and 0.5 pertain to liquid fossil fuel combustion sources, and ratios greater than 0.5 are linked to coal or biomass combustion sources. Pyr/BaA ratios of less than 2 suggest coal or biomass combustion sources, while ratios between 2 and 6 indicate liquid fossil fuel combustion sources [48].

#### 2.7.2. Positive Matrix Factorization Method (PMF)

Paatero and Tapper proposed positive matrix factorization (PMF) in 1993 [49]. Weights were employed to calculate the error associated with each chemical component in the particulate matter. The least squares method was utilized to identify the primary sources and their contributions [50]. PMF does not require the measurement of source component spectra, incorporates non-negative elemental sharing in the decomposition matrix, utilizes the standard deviation of the data for optimization, and is capable of handling missing and imprecise data. This is represented in the following equation:

(6) $$X_{ij}=\sum_{k=1}^{p}{(G}_{ik}\times F_{kj})+E_{ij},$$

(7) $$Q=\sum_{i=1}^{n}\sum_{j=1}^{m}{\left(\frac{X_{ij}-\sum_{k=1}^{p}G_{ik}{\times F}_{kj}}{U_{ij}}\right)}^{2}$$

In this context, the equation represents the concentration of species j in the i-th PAH sample, the contribution of the k-th source in the i-th sample, the fraction of species j in the k-th source, and the error associated with species j in the i-th sample. As outlined in the user manual produced by the EPA, the method detection limit (MDL) and the error factor are utilized in processing the resulting detection concentration data. If data are absent, the corresponding entry is removed. When the detection concentration is equal to or less than the MDL, it is set to 1/2 MDL, with 5/6 MDL serving as the uncertainty value. Conversely, if the detection concentration exceeds the MDL, the observed value is used as the detection concentration. The following EPA-recommended Formula (9) was employed to calculate uncertainty:

(8) $$U_{ij}=\sqrt{{\left(Errorfraction+c\right)}^{2}+{\left(0.5\times MDL\right)}^{2}}$$

## 3. Results and Discussion

### 3.1. Pollution Level and PAHs Locations

**Figure 2:**
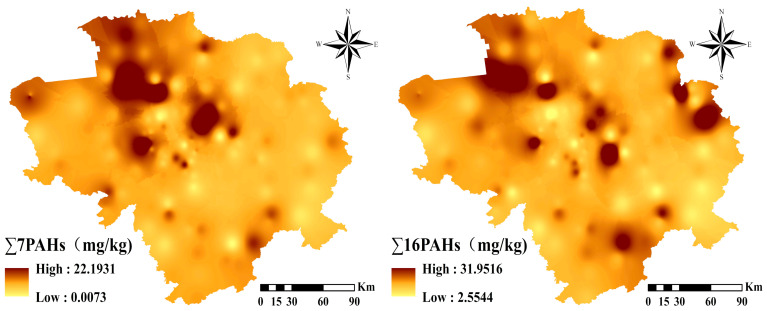
Total content profiles of 7 carcinogens and 16 PAHs in black soil region of Jilin Province.

**Figure 3:**
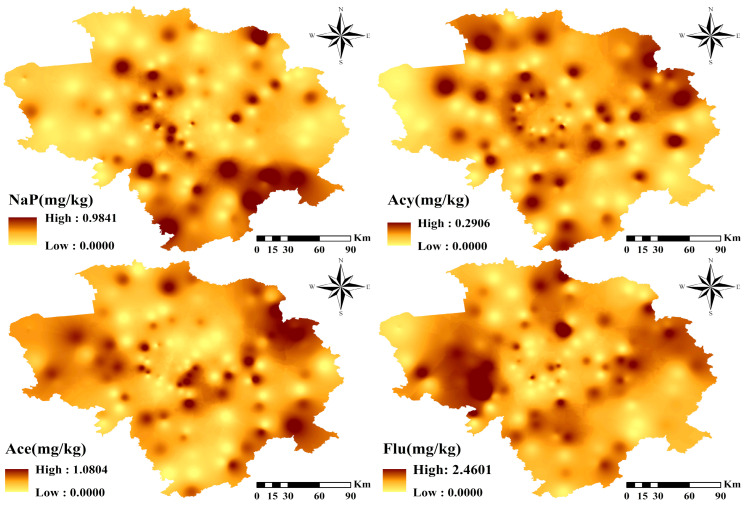

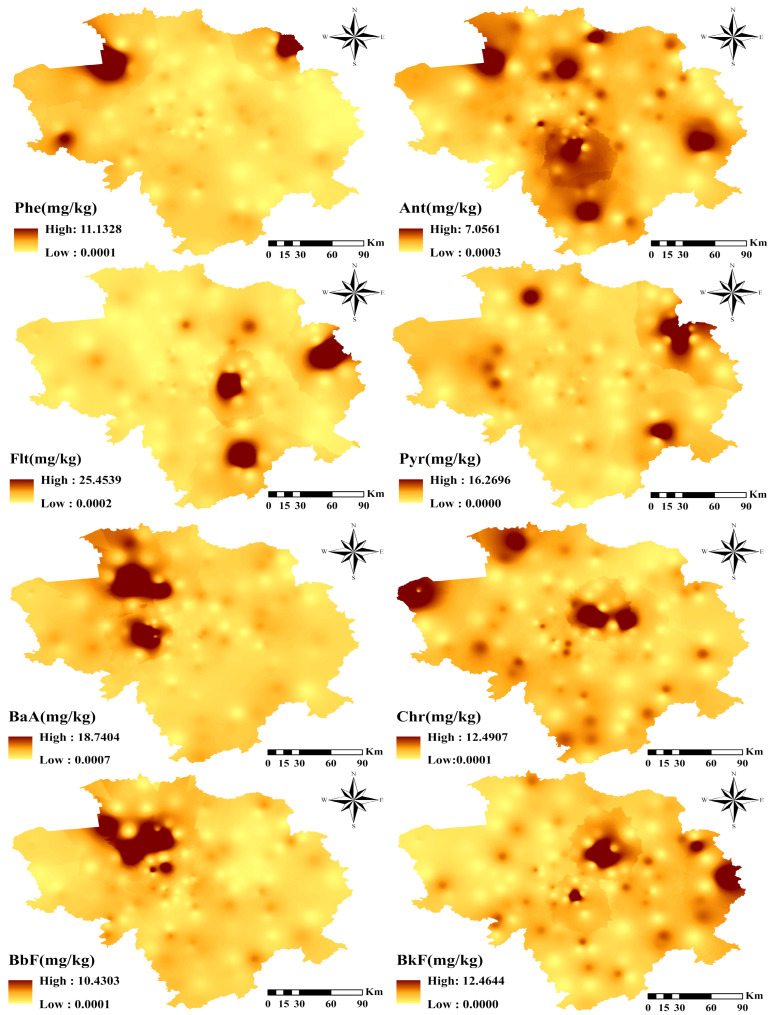

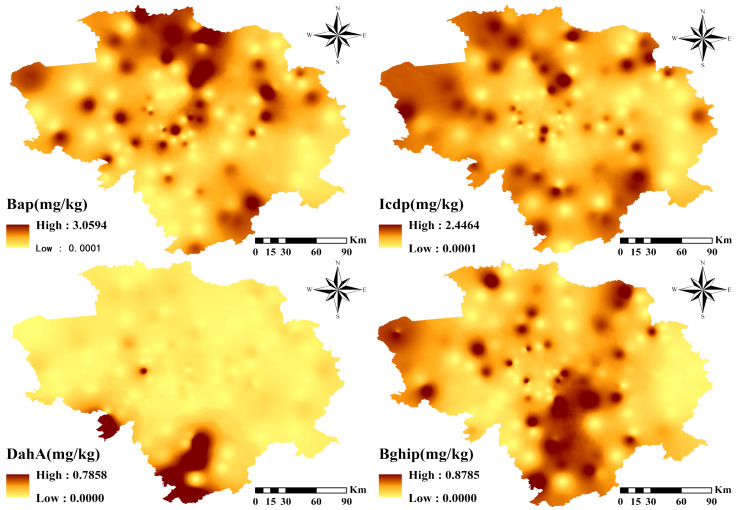
Spatial distribution of 16 PAHs in black soil region of Jilin Province.

Figure 2 and Figure 3 and Table 1 show the concentration levels of 16 PAHs in Jilin Province soils. The mean values were 0.07 and 1.19 mg/kg, with the highest concentrations as follows (unit: mg/kg): BaA (1.19) > Flt (1.08) > Chr (0.95) > Pbe (0.79) > Pyr (0.76) > Ant (0.74) = IND (0.74) > BaP (0.69) > BkF (0.68) > Flu (0.63) > BbF (0.55) > Ace (0.44) > BghiP (0.27) > Nap (0.26) > Acy (0.11) > DBahA (0.07). The mean values of BaA and Flt were over 1 mg/kg, and the mean value of DBahA was less than 0.01 mg/kg. The 16 PAHs ranged from 0 to 25.45 mg/kg, with an average of 0.62 mg/kg. The 7 carcinogenic PAHs ranged from 0 to 18.74 mg/kg, with a mean of 0.7 mg/kg. PAH levels vary significantly and some sites are contaminated. The number of benzene rings affects the properties of a PAH, with 2–3-ring PAHs being volatile and PAHs with 4 rings or more are not [51]. The study found that 2–6-ring PAHs made up 2.65%, 27.22%, 39.97%, 26.71% and 3.45% of the total, respectively, with 4–6-ring PAHs being relatively high. LMW PAHs ranged from 0.250 mg/kg to 16.346 mg/kg with a mean of 2.973 mg/kg, while HMW PAHs ranged from 1.689 mg/kg to 30.327 mg/kg with a mean of 6.997 mg/kg (Table 1).

Table 1 presents the levels of PAHs from various cities around the world. PAHs pose a risk to human health when inhaled. Current research on large and medium-sized PAHs in China primarily focuses on atmospheric particles and pollution characteristics, with increasing attention being directed towards agricultural soils. Table 2 illustrates the PAH levels in different regions globally. The PAH concentrations reported in this study are significantly higher than those found in other Chinese cities, including Huanghuai, Shanghai, Xinzhou, the Junggar Basin, Fujian, Yichang, and Beijing. When compared with international cities, the levels are greater than those in Delhi, King George Island, Australia, South Korea, Japan, Switzerland, West Palm Beach, and the Lesser Himalayas, yet lower than those observed in London. Jilin Province serves as a crucial industrial and agricultural hub in China and is recognized as the birthplace of the automotive and chemical industries. The rapid development of Jilin Province has resulted in elevated PAH levels in the soil of industrial areas and urban centers compared with other urban regions. This indicates a potential contamination of the soil in Jilin Province.

**Table 2:** PAH levels in soil worldwide (mg/kg).

Placements	Soil Type	Total Concentration Range	Mean	Cited Material
Huanghuai, China	Agricultural soils	0.0157–1.2476	0.1295	[52]
Shanghai, China	Agricultural soils	0.0223–8.214	1.552	[36]
Xinzhou, China	Agricultural soils	0.00–0.782	0.202	[53]
Junggar Basin, Xinjiang	Agricultural soils	0.0139–0.1974	0.0455	[54]
Fujian, China	Agricultural soils	0.0129–2.271	0.2239 ± 0.3243	[55]
Yichang, China	Surface soils	0.0083–0.397	0.0558	[56]
Shanghai, China	Agricultural soils	0.0172–3.775	0.339 ± 0.594	[57]
Changbai Mountain range, China	Mountain soil	0.239–2.433	1.047	[58]
Beijing, China	Urban soil	0.066–6.867	0.460	[59]
London, UK	Urban soils	4.000–67.000	18.000	[60]
Delhi, India	Agricultural soils	0.830–3.880	1.910 ± 1.020	[61]
Lucknow, India	Urban soils	0.4789–8.1641	3.7482	[62]
King GeorgeIsland, Antarctica	Surface soils	0.0018–0.0329	0.0119 ± 0.0081	[63]
Australia	Industrial soils	0.0529–6.2401	0.7514	[64]
South Korea	Agricultural soils	0.0223–2.834	0.236	[65]
Japan	Agricultural soils	0.0046–2.000	0.465	[66]
Switzerlan	Agricultural soils	0.05–0.619	0.225	[67]
West PalmBeach, USA	Urban soils	0.922–17.698	4.055	[68]
Lesser himalayan, Pakistan	Surface soils	0.0145–0.4374	0.492	[69]

The total concentration of 16 PAHs in soil samples from agricultural fields in Jilin Province was assessed using the methodology proposed by Maliszewska-Kordybach. According to this method, a concentration of less than 0.2 indicates non-contamination, 0.2 to 0.6 signifies weak contamination, 0.6 to 1 denotes contamination, and concentrations exceeding 1 indicate heavy contamination [70]. Based on Maliszewska-Kordybach’s classification criteria, the agricultural soils in Jilin Province are heavily contaminated with PAHs. Therefore, further investigation into the agricultural soils of Jilin Province is warranted.

### 3.2. Correlation Analysis Between PAHs and Soil Properties

**Figure 4:**
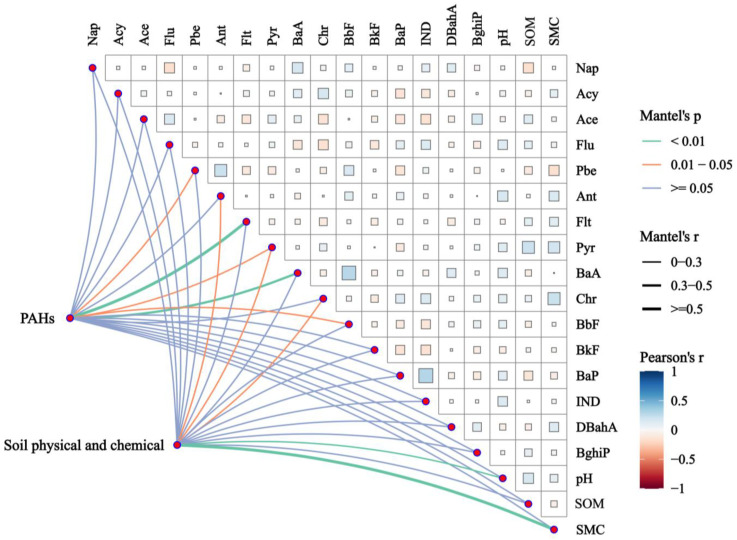
Correlation analysis of PAH concentration with pH, soil organic matter and soil moisture content.

Correlation analyses facilitate the identification of relationships between various targets. Factors such as soil texture, pH, microbial activity, and crop types significantly influence the persistence of PAHs in the soil. In the study area, soil pH ranged from 3.9 to 8.14, with a mean value of 5.74. The moisture content of the soil (SMC) varied between 6.2% and 48.6%, resulting in a mean value of 22.6%. Soil organic matter (SOM) was found to range from 0.66% to 6.72%, with an average of 1.91%. According to the correlation analysis of PAH concentration and soil physicochemical properties (Figure 4), the majority of HMW PAHs were positively correlated with soil pH, SOM and SMC, and there were some effects between soil physico-chemical properties and HMW PAHs. Researcher found that soil PAHs were influenced by various soil properties [71]. HMW PAHs were particularly linked to soil pH, SOM, and moisture levels. Additionally, some correlations were observed between soil properties and HMW PAHs. SOM plays a crucial role in the behavior of PAHs in soil, as PAHs are absorbed by soil organic matter; the greater the absorption, the less available they are for uptake by living organisms [72]. The presence of HMW PAHs in soil was associated with SOM, which may be influenced by the interactions among hydroxyl, carboxyl, and carbon functional groups in SOM and PAHs, thereby facilitating the retention of anthropogenic PAHs [73]. Furthermore, soil pH affects the adsorption capacity of SOM.

### 3.3. Analysis of the PAH Sources

#### 3.3.1. Feature Ratio Method Source Analysis

**Figure 5:**
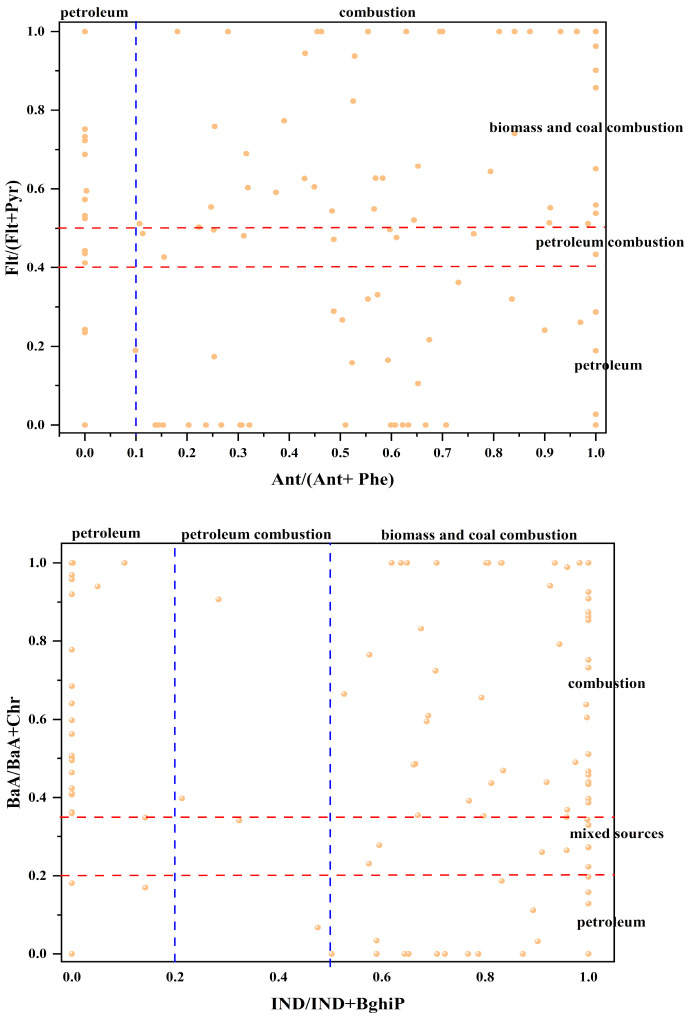
Source analysis results of PAHs in black soil of Jilin Province.

Understanding the origins of PAHs in soil is crucial for assessing and managing pollution. The isomeric ratio method is an effective approach for analyzing PAH sources, as different pollution sources exhibit distinct ratio characteristics. In Jilin Province, the average concentrations of PAHs in agricultural soils were found to be 2.973 mg/kg for LMW PAHs and 6.977 mg/kg for HMW PAHs, resulting in a ratio of 0.42. This indicates that combustion sources are the primary contributors to PAHs in the surface soils of the study area. The Ant/(Ant + Phe) ratio serves to differentiate between PAHs derived from petroleum and those from combustion sources. Furthermore, the Flt/(Flt + Pyr), BaA/(BaA + Chr), and IND/(IND + BghiP) ratios are employed to distinguish between petroleum, liquid fossil fuel combustion, and coal/biomass combustion sources. The ratios of Flt to Pyr, IND, and BghiP are considered stable and reliable. In this study, Ant/(Ant + Phe) was plotted as the x-axis against Flt/(Flt + Pyr) on the y-axis; similarly, IND/(IND + BghiP) was plotted against BaA/(BaA + Chr). Scatter plots were utilized to analyze the sources of PAHs in the surface soil of the study area (Figure 5).

The ratio of Ant/(Ant + Phe) exceeded 0.1 in 75.6% of the samples, indicating that the PAHs in the surface layer of soil in Jilin Province predominantly originate from pyrolysis sources. Additionally, the ratio of Flt/(Flt + Pyr) was greater than 0.5 in 48.7% of the samples, while 2% of the samples recorded values below 0.4, suggesting that the PAHs in the soil are influenced by a combination of petroleum sources and coal/biomass. The combustion source influenced 68.1% of the BaA/(BaA + Chr) ratios and 65.5% of the IND/(IND + BghiP) ratios. The varying ratios of different PAH molecules across the four groups indicate that the sources of PAHs in the surface soils of agricultural fields in Jilin Province are quite complex. Specifically, LMW PAHs are primarily derived from oil, whereas HMW PAHs are associated with combustion processes. Consequently, oil and combustion emerge as the principal sources of PAHs in the environment. Most sampling sites are located near streets and motorways and are influenced by overpasses and vehicle emissions. Furthermore, industrial activities in Jilin Province also contribute to the levels of PAHs. The presence of petroleum sources at the sampling sites is significant, as PAHs are constituents of petroleum. The four diagnostic ratios exhibited notable differences between sources and receptors, highlighting the need for further source identification.

#### 3.3.2. PMF Source Analysis

**Figure 6:**
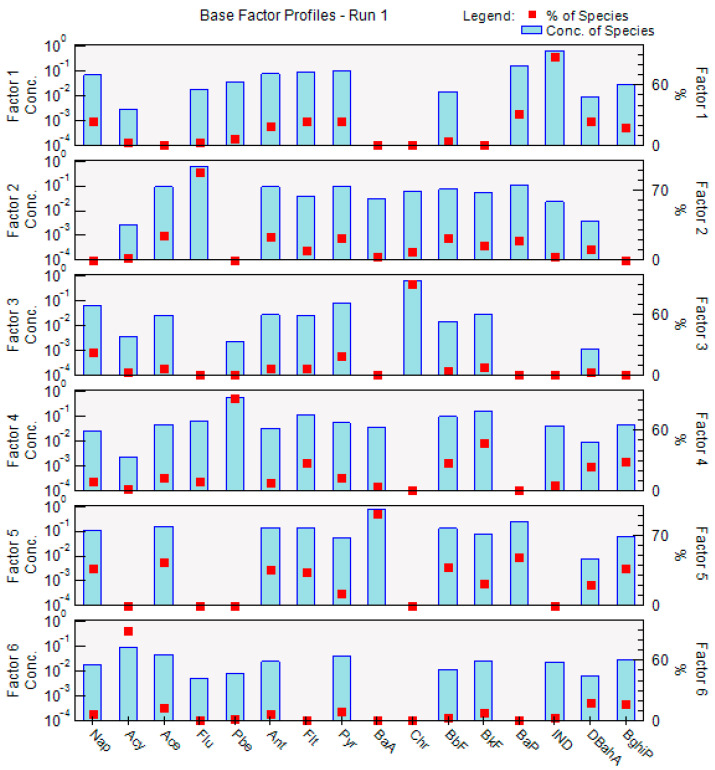
The PMF source analysis results for 16 PAHs in black soil of Jilin Province.

To control farmland pollution in Jilin Province, it is essential to better understand the sources of soil pollutants. This study employed the EPA PMF5.0 model to analyze PAH contamination in the region. The analysis incorporated the concentrations and uncertainties of 16 PAHs, classified as ’strong.’ The base model was executed 20 times, with the first run yielding the most favorable results, characterized by the smallest Q value and by residual values ranging between −3 and 3. The predicted-to-observed values were approximately equal to 1, indicating the best fit. The PMF model identified six distinct factors, with the results shown in Figure 6.

The primary contributors to Factor 1 were IND, Pyr, and DBahA, which accounted for 88.3%, 24.1%, and 23.4%, respectively. IND contributed the most, and both IND and DBahA were associated with the traffic source, as demonstrated in previous studies [74]. Consequently, Factor 1 is identified as the traffic source. Factor 2 is predominantly caused by Flu, which is a product of industrial boilers and cogeneration plants [75], indicating that Factor 2 represents an industrial emission source. The principal contributor to Factor 3 is Chr, with a contribution rate of 94.2%. This predominance arises from the significant production of Chr when coal is heavily loaded into the combustion profile [76]. In Jilin Province, coal serves as the primary energy source for warm air supply, thus establishing Factor 3 as coal combustion. Factor 4 was primarily influenced by Phe and BkF, contributing 92.4% and 46.7%, respectively. Phe is derived from petroleum and diesel vehicles, while BkF is released from diesel vehicles [77], indicating that Factor 4 originates from diesel/oil combustion. The main contributors to Factor 5 include BaA, Bap, Ace, BbF, Nap, BghiP, Ant, Flt, and BkF, with contributions of 92%, 48.3%, 43.2%, 38.3%, 37.5%, 36.8%, 36.2%, 33.8%, and 22.4%, respectively. This factor was predominantly characterized by HMW PAHs. BaA and Flt are recognized as markers of coal combustion, while Bap, BbF, BkF, and BghiP are indicative of petroleum combustion, particularly from diesel and petrol sources [78]. Ant, Nap, and Ace serve as markers of coke production [74]. Therefore, Factor 5 is primarily associated with coal, oil, and transportation. Acy emerged as the main contributor to Factor 6, accounting for 60%. Acy is indicative of wood combustion [79], thereby categorizing Factor 6 as the incomplete combustion of biomass, such as wood.

Jilin Province is a region characterized by significant industrial activity, which has contributed to both economic growth and environmental pressure on the soil. The study area experiences elevated levels of organic pollutants in the air, primarily due to the reliance on coal and firewood for heating in rural areas of the northeast. This reliance generates straw and other agricultural waste [80]. Additionally, the region is impacted by vehicle exhaust, industrial emissions, and mining activities, as well as the extraction and use of minerals. Researchers have identified combustion as a significant contributor to the presence of polycyclic aromatic hydrocarbons (PAHs) [81], which includes emissions from coal combustion for industrial purposes and winter heating, as well as oil combustion in automobile engines.

Researchers have conducted a study on pollution in three representative industries—petrochemical, machinery manufacturing, and non-ferrous smelting—within the Northeast Heavy Industrial Zone (NHIZ) [82]. Their findings indicate that the petrochemical industry exhibited the highest levels of PAHs, characterized by the most complex pollution status and diverse sources of contamination. Furthermore, the researchers report that, over a span of 20 years, the primary sources of PAHs in both industrial and rural areas transitioned from coal and oil to include coking, coal and biomass combustion, as well as vehicle emissions and biomass burning [13]. The PMF analyses of PAHs in Jilin soils revealed that coal, oil, and traffic constitute the main local sources of pollution.

### 3.4. Human Health Risk Assessment

**Figure 7:**
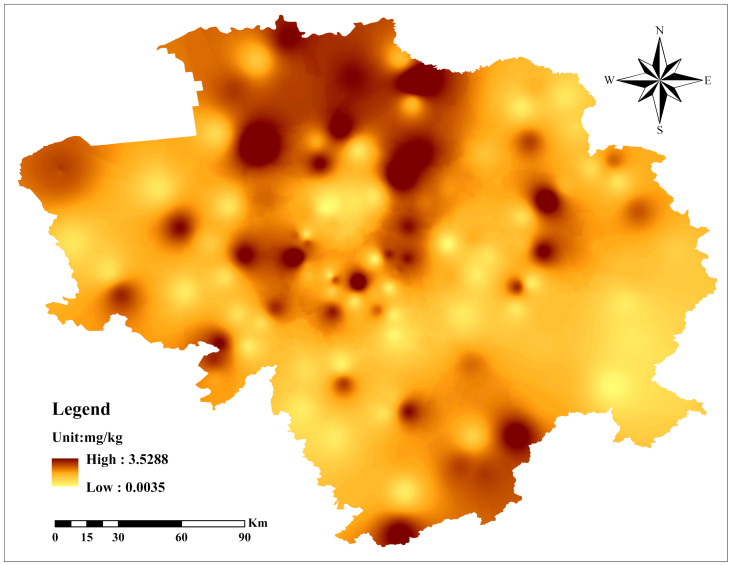
TEQ_BaP_ level distribution in the black soil region of Jilin Province.

PAHs in black soil may be toxic to humans, so we calculated the TEQ_BaP_ value of PAHs in soil using a formula (Table 3 and Figure 7). The TEQ of single PAHs ranged from 0 to 3.0739 mg/kg and the mean size is as follows: BaP (0.6888) > BaA (0.1191) > IND (0.0744) > DBahA (0.0694) > BkF (0.0677) > Chr (0.0095) > Ant (0.0074) > BghiP (0.0027) > Flt (0.0011) > Phe (0.0008) = Pyr (0.0008) > Flu (0.0006) > Ace (0.0004) > Nap (0.0003) > Acy (0.0001), which had the highest content of BaP and BaA. The TEQ_BaP_ of PAHs ranged from 0.0025 to 3.5448 mg/kg, with a mean of 1.0979 mg/kg. Figure 7 shows the horizontal distribution of TEQ_BaP_ levels in Jilin Province. In 2010, the Canadian Council of Environment Ministers (CCME) issued guidelines recommending that TEQ_BaP_ levels below 0.7 mg/kg are safe [83]. This study found that TEQ_BaP_ was a low risk, indicating that PAHs in black soil farmland in Jilin Province may cause cancer.

**Figure 8:**
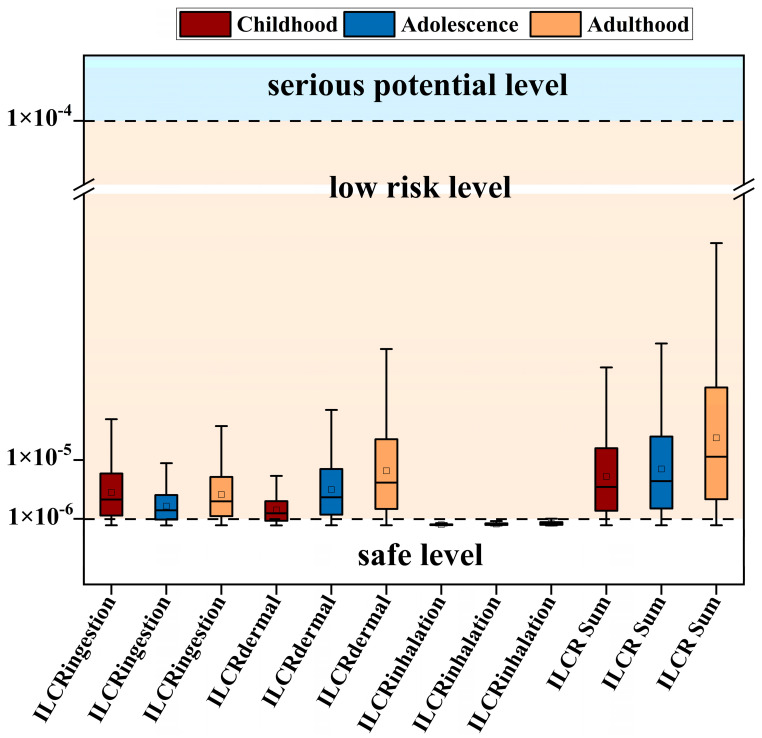
Carcinogenic risk of PAHs in different exposure routes in Jilin Province.

The ILCR model illustrates the carcinogenic risk of PAHs in Jilin Province across various exposure routes (Figure 8). The ILCR_ing_ values for children ranged from 1.68 × 10^−8^ to 1.63 × 10^−5^; for youth, from 6.84 × 10^−9^ to 9.52 × 10^−6^; and for adults, from 1.09 × 10^−9^ to 1.52 × 10^−5^. The size is as follows: children (average: 5.03 × 10^−6^) > adults (average: 4.71 × 10^−6^) > youth (average: 2.95 × 10^−6^). ILCR_der_ for children is between 5.44 × 10^−9^ and 7.58 × 10^−5^; for young people, between 1.27 × 10^−8^ and 1.77 × 10^−5^; and for adults, between 1.94 × 10^−8^ and 2.70 × 10^−5^, with sizes as follows: adult (mean: 8.36 × 10^−6^) > youth (mean: 5.47 × 10^−6^) > child (mean: 8.36 × 10^−6^). The ILCR_inh_ in children ranged from 2.47 × 10^−10^ to 3.44 × 10^−7^; in youth, from 4.69 × 10^−10^ to 6.53 × 10^−7^; and in adults, from 7.41 × 10^−10^ to 1.03 × 10^−6^. The average risk levels are as follows: adults (average: 3.19 × 10^−7^) > youth (average: 2.02 × 10^−7^) > children (average: 1.06 × 10^−7^).

The intake pathway of PAH was analyzed. Children with values ranging from 1.74 × 10^−8^ to 2.42 × 10^−5^ exhibited an average value of 7.49 × 10^−6^. Youth within the range of 2.00 × 10^−8^ to 2.78 × 10^−5^ had a mean value of 8.62 × 10^−6^. The overall range observed was from 3.10 × 10^−8^ to 4.32 × 10^−5^, with a mean value of 1.34 × 10^−5^. The highest values for intake, skin contact, and inhalation routes were noted in the adult group. The safe pollution level is defined as an ILCR value of less than 10^−6^; values between 10^−6^ and 10^−4^ indicate a lower risk, while values greater than 10^−4^ signify a higher risk [45]. In this study, the assessed risks were also considered low.

Table 4 presents the ILCR values of PAHs across various regions worldwide in recent years. Children face a higher risk of ILCR in Sichuan and Chongqing compared with other areas, but they are at a lower risk than those in Chengdu, Dongying, and Nigeria. Youth exhibit a lesser risk than those in Chengdu. Adults are at a lower risk than individuals in Sichuan, Chongqing, Chengdu, Dongying, and Nigeria. Overall, PAHs do not pose a significant threat to the local population; however, the risk increases with the age of the population. In Jilin Province, cancer among adults is a concern. To mitigate this risk, it is advisable to increase the buffer zone between farmland and residential areas, enhance farm management practices, and implement innovative pollution prevention and control strategies.

### 3.5. Uncertainly Analysis

Based on data from 119 sampling points in the black soil of Jilin Province, we evaluated PAH pollution levels and ecological risks and developed a model correlating pollution source activities with cumulative soil pollutant concentrations. While our model is grounded in classical theoretical approaches, uncertainties remain due to treating overall production and lifestyle in Jilin Province as a single large-scale pollution source and challenges in classifying pollutant origins due to data limitations. Future research could address these uncertainties by conducting detailed analyses of specific pollution sources over time and by establishing industry specific inventories of raw material usage and pollution emissions. This would enhance predictions of pollutant concentrations and risk assessments, facilitating more targeted preventive measures. This approach aims to refine our model and provide a stronger basis for environmental management.

## 4. Conclusions

In this study, we analyzed the concentration, source and spatial distribution of PAHs in the black soil area of Jilin Province. The concentration of ∑16 PAHs in the study area ranged from 2.546 to 33.993 mg/kg, and the highest proportion of HMW PAHs was found among PAHs with 2–6 rings, accounting for 70.13%. A two-source analysis of the eigenvalue ratio method and the PMF model revealed that human activities, including coal and biomass combustion, traffic pollution, and industrial pollution, are the primary sources of soil PAH contamination. These sources are largely associated with local coal burning for heating, automobile exhaust emissions, straw burning, factory pollutant emissions, and other human activities. The TEQ_BaP_ of ∑16PAHs ranged from 0.0025 to 3.5448 mg/kg, with a mean value of 1.0979 mg/kg. These values were combined with the age structure of the population to calculate the precise ILCRs, revealing that the highest values were associated with the ingestion, dermal contact, and inhalation routes in the adult group. Specifically, the calculated ILCR values for adults (1.34 × 10^−5^) exceeded those for adolescents (8.62× 10^−6^) and children (7.49 × 10^−6^). This finding raises concerns regarding the elevated cancer risk among adults in Jilin Province. Consequently, it is recommended that measures be implemented to mitigate this risk, including increasing the isolation zone between farmland and residential areas, enhancing the management system, and adopting advanced pollution prevention and control technologies. The findings of this study provide valuable insights into the exposure of PAHs in agricultural soils in Jilin Province, which can inform strategies to mitigate PAH contamination in soil and promote sustainable agricultural development in the region.

## Figures and Tables

**Table 1:** Polycyclic aromatic hydrocarbons in Jilin Province farmland soil (mg/kg).

PAHs	Aromatic Ring	TEF ^1^	Min	Max	Mean	Median	SD	Skewness	Kurtosis
Nap	2	0.001	0.000	0.984	0.264	0.178	0.272	0.764	−0.589
Acy	3	0.001	0.000	0.291	0.111	0.099	0.099	0.301	−1.342
Ace	3	0.001	0.000	1.080	0.436	0.419	0.359	0.189	−1.348
Flu	3	0.001	0.000	1.609	0.628	0.574	0.539	0.646	0.351
Phe	3	0.001	0.000	2.460	0.790	0.607	1.463	6.134	41.909
Ant	3	0.01	0.000	11.163	0.744	0.587	0.928	3.417	18.389
Flt	4	0.001	0.000	25.454	1.082	0.531	3.129	6.220	41.103
Pyr	4	0.001	0.000	16.270	0.756	0.578	1.603	8.050	75.899
BaA	4	0.1	0.000	18.740	1.191	0.727	2.337	5.736	36.359
Chr	4	0.01	0.000	12.491	0.948	0.606	1.487	4.984	33.336
BbF	5	0.1	0.000	10.430	0.548	0.328	1.31	6.512	51.892
BkF	5	0.1	0.000	12.464	0.677	0.316	1.381	6.364	49.179
BaP	5	1	0.000	3.074	0.689	0.455	0.77	0.874	−0.158
IND	5	0.1	0.000	2.446	0.744	0.616	0.677	0.362	−1.186
DBahA	6	1	0.000	0.786	0.069	0.026	0.157	3.670	12.804
BghiP	6	0.01	0.000	0.879	0.274	0.139	0.306	0.729	−0.982
LMW PAHs ^2^			0.250	16.346	2.973	2.714	1.978	4.040	22.943
HMW PAHs ^3^			1.689	30.327	6.977	5.994	4.660	2.568	7.856
∑7Car PAHs ^4^			0.000	22.316	4.866	4.288	3.466	2.952	11.281
∑16PAHs ^5^			2.546	31.993	9.950	9.057	4.863	2.089	5.729

SD: standard deviation; ^1^: PAH toxicity factor compared with BaP; ^2^: low molecular weight PAHs; ^3^: high molecular weight PAHs; ^4^: concentrations of 7 carcinogenic PAHs; ^5^: concentrations of 16 PAHs.

**Table 3:** TEF and TEQ_BaP_ for Jilin Province ∑16PAHs.

PAHs	TEFi	Min	Max	Mean	PAHs	TEFi	Min	Max	Mean
Nap	0.001	0.000	0.0010	0.0003	BaA	0.1	0.000	1.8740	0.1191
Acy	0.001	0.000	0.0003	0.0001	Chr	0.01	0.000	0.1249	0.0095
Ace	0.001	0.000	0.0011	0.0004	BbF	0.1	0.000	1.0430	0.0548
Flu	0.001	0.000	0.0025	0.0006	BkF	0.1	0.000	1.2464	0.0677
Phe	0.001	0.000	0.0112	0.0008	BaP	1.00	0.000	3.0739	0.6888
Ant	0.010	0.000	0.0706	0.0074	IND	0.10	0.000	0.2446	0.0744
Flt	0.001	0.000	0.0255	0.0011	DBahA	1.00	0.000	0.7858	0.0694
Pyr	0.001	0.000	0.0163	0.0008	BghiP	0.010	0.000	0.0088	0.0027

**Table 4:** Average ILCR and ILCRs for children and adults across the world.

Site	Description	ILCR_ing_	ILCR_der_	ILCR_inh_	ILCR_s_	Reference
Sichuan, China	Children	6.08 × 10^−9^	2.44 × 10^−12^	7.58 × 10^−8^	8.19 × 10^−8^	[84]
	Adult	7.78 × 10^−9^	3.12 × 10^−12^	1.38 × 10^−5^	1.38 × 10^−5^	
Chongqing, China	Children	7.08 × 10^−9^	2.84 × 10^−12^	8.83 × 10^−8^	9.53 × 10^−8^	[84]
	Adult	9.06 × 10^−9^	3.63 × 10^−12^	1.61 × 10^−5^	1.61 × 10^−5^	
Chengdu, China	Children	8.05 × 10^−6^	5.84 × 10^−6^	1.56 × 10^−6^	1.39 × 10^−5^	[83]
	Adolescents	7.95 × 10^−6^	6.53 × 10^−6^	8.07 × 10^−6^	1.46 × 10^−5^	
	Adult	8.88 × 10^−6^	8.95 × 10^−6^	1.39 × 10^−6^	1.79 × 10^−5^	
Dongying, China	Children	4.76 × 10^−7^	1.05 × 10^−5^	3.48 × 10^−5^	4.58 × 10^−5^	[85]
	Adult	6.78 × 10^−7^	3.13 × 10^−5^	1.37 × 10^−5^	1.73 × 10^−4^	
Nigeria, Africa	Children	2.95 × 10^−2^	3.68 × 10^−3^	5.73 × 10^−3^	6.42 × 10^−4^	[86]
	Adult	2.31 × 10^−3^	4.10 × 10^−3^	1.79 × 10^−7^	6.19 × 10^−4^	

## Data Availability

Data are contained within the article.

## Abbreviations

PAHs	Polycyclic aromatic hydrocarbons
PMF	Positive matrix factorization method
ILCR	Incremental lifetime cancer risk
TEFs	Toxicity equivalence factors
TEQ_BaP_	Toxic equivalent concentration
Nap	Naphthalene
Acy	Acenaphthylene
Ace	Acenaphthene
Flu	Fluorene
Phe	Phenanthrene
Ant	Anthracene
Flt	Fluoranthene
Pyr	Pyrene
BaA	Benz(a)anthracene
Chr	Chrysene
BbF	Benzo(b)fluoranthene
BkF	Benzo(k)fluoranthene
BaP	Benzo(a)pyrene
IND	Indeno (1,2,3-cd) pyrene
DBahA	Dibenz (a,h)anthracene
BghiP	Benzo (g,h,i)perylene
